# Synergistic Effect of SRY and Its Direct Target, WDR5, on Sox9 Expression

**DOI:** 10.1371/journal.pone.0034327

**Published:** 2012-04-16

**Authors:** Zhen Xu, Xinxing Gao, Yinghong He, Junyi Ju, Miaomiao Zhang, Ronghua Liu, Yupeng Wu, Chunyan Ma, Chi Ma, Zhaoyu Lin, Xingxu Huang, Quan Zhao

**Affiliations:** 1 The State Key Laboratory of Pharmaceutical Biotechnology, Molecular Immunology and Cancer Research Center, School of Life Sciences, Nanjing University, Nanjing, China; 2 Model Animal Research Center, Nanjing University, Nanjing, China; 3 School of Basic Medicine, Dali University, Yunnan, China; Shantou University Medical College, China

## Abstract

SRY is a sex-determining gene that encodes a transcription factor, which triggers male development in most mammals. The molecular mechanism of SRY action in testis determination is, however, poorly understood. In this study, we demonstrate that WDR5, which encodes a WD-40 repeat protein, is a direct target of SRY. EMSA experiments and ChIP assays showed that SRY could bind to the WDR5 gene promoter directly. Overexpression of SRY in LNCaP cells significantly increased WDR5 expression concurrent with histone H3K4 methylation on the WDR5 promoter. To specifically address whether SRY contributes to WDR5 regulation, we introduced a 4-hydroxy-tamoxifen-inducible SRY allele into LNCaP cells. Conditional SRY expression triggered enrichment of SRY on the WDR5 promoter resulting in induction of WDR5 transcription. We found that WDR5 was self regulating through a positive feedback loop. WDR5 and SRY interacted and were colocalized in cells. In addition, the interaction of WDR5 with SRY resulted in activation of Sox9 while repressing the expression of β-catenin. These results suggest that, in conjunction with SRY, WDR5 plays an important role in sex determination.

## Introduction

WDR5, also named BIG-3, was first identified in a murine prechondroblastic cell line by differential display PCR following induction with BMP-2. Analysis of liver, spleen, kidney, and other tissues showed that the highest level of WDR5 expression was in testis [1]. WDR5 is a member of the WD-40 repeat protein family, which exhibits a seven-bladed propeller-like structure with a narrow channel running through the center [2], [3].

In the last several years,studies have focused on the role of WDR5 associated with MLL and SET1 complexes, which trigger methylation of histone H3K4. WDR5 is the core component of the MLL/SET1 complex, and it is indispensable for assembly and effective methyltransferase activity of the complex [2]. It was shown that WDR5 interacts with histone H3 regardless of the methylation status of the Lysine 4 residue [2]. WDR5 regulates osteoblast differentiation and vertebrate development [4], [5]. In addition, WDR5 has been shown to be a regulator of embryonic stem cell self-renewal, and its expression correlates with the undifferentiated state [6]. However, it is not known how WDR5 itself is regulated.

SRY is a testis-determining gene located on the Y-chromosome, which triggers male development in most mammalian embryos. Mutations in SRY are associated with human XY gonadal dysgenesis [7]. The open reading frame (ORF) of human SRY contains only a single exon and encodes a 204-amino-acid protein, which is composed of three regions: a central 79 amino acids HMG domain, C-terminal domain, and N-terminal domain. The HMG domain, which is highly conserved between species, binds sites in the minor groove of DNA and introduces local conformational changes that influence transcription of genes downstream [8]–[10]. SRY, a member of the Sox (SRY-related HMG box) gene family, interacts with CaM (calmodulin) and importin β and facilitates translocation of proteins from cytoplasm to nucleus [11], [12]. Unlike mouse, human SRY lacks a C terminal transcription activation domain necessary for male sex determination, suggesting that human SRY may function through interaction with additional transcriptional co-activators. In vitro analysis of recombinant SRY protein suggests that it recognizes a degenerate motif (A/T)AACAA(A/T), making it difficult to identify its in vivo targets [13]. To date, few regulatory target genes of SRY have been identified; these include Sox9, Cbln4, TCF21, and NTF3 [14]–[17]. The molecular mechanism of SRY action in testis determination is poorly understood [18], [19].

In this study, we show that WDR5 is a direct target of SRY. The interaction of WDR5 and SRY activates Sox9 expression. As Sox9 is the master regulator of sex determination [18], [19], we hypothesize that WDR5 interacts with SRY to promote testis development.

## Results

### Identification of potential SRY binding sites in the proximal WDR5 promoter

The human WDR5 gene has two transcript variants, which encode the same protein but differ in the 5′UTR (Fig. S1A). Alignment of the proximal promoter of the WDR5 gene (between −2000 bp and +1 bp) in different vertebrates reveals that the proximal promoter of Variant 2 (the shorter variant) is more conserved (Fig. S1B). Thus, in this study, we focused on the Variant 2 promoter to investigate transcriptional regulatory mechanisms of WDR5.

**Figure 1:**
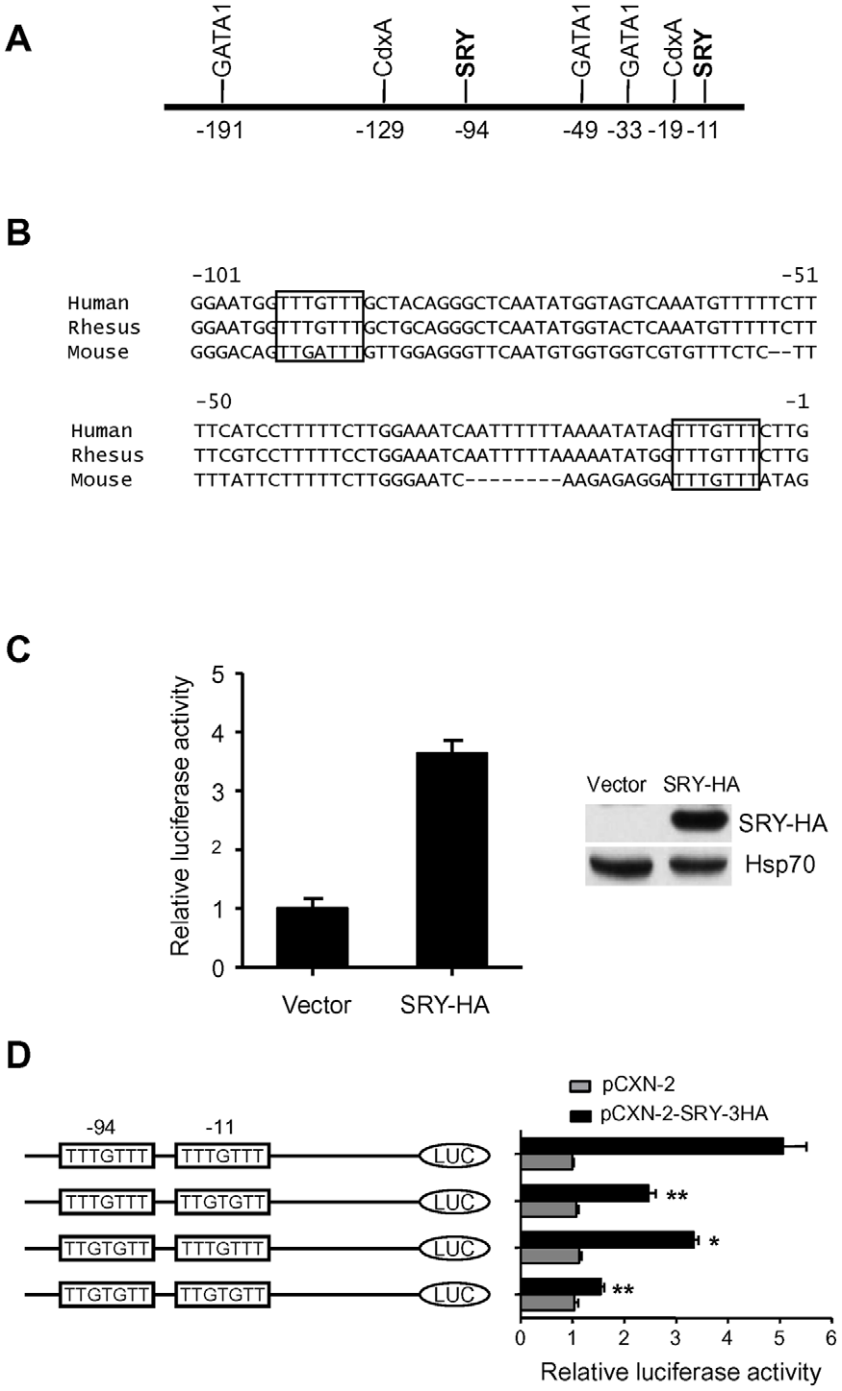
Identification of potential SRY binding sites in WDR5 proximal promoter. (A) Schematic representation of WDR5 proximal promoter with two potential SRY binding sites and GATA1 and CdxA binding sites. (B) Alignment of WDR5 proximal promoters between human, rhesus, and mouse. Regions in frame represent the SRY binding sites. (C) Relative luciferase activity assay from cells containing either vector or overexpressing SRY. Graphs show mean ± SD, n = 3 (left panel). Western blot analysis of HA-tagged SRY and loading control, Hsp70 (right panel). (D) Relative luciferase activity assay from cells containing either wild type promoter or mutant promoters with either vector or HA tagged SRY. Graphs show mean ± SD, n = 3. *P<0.05, **P<0.01 compared to wild type (top panel); Student's t-test.

To predict potential transcription factors, we used the TF-SEARCH program (http://www.cbrc.jp/research/db/TFSEARCH.html) to search a 2.0-kb fragment of genomic DNA that contains part of the first exon of WDR5 and 5′UTR. The result revealed that within the proximal 200 bp promoter region several transcription factors were likely to bind, including CdxA, GATA-1, and SRY (Fig. 1A). Notably, there were two predicted SRY binding sites in this region (sequence: TTTGTTT), which were exactly complementary to the SRY binding consensus sequence (A/T)AACAA(A/T). These sites were further checked using ConSite (http://mordor.cgb.ki.se/cgibin/CONSITE/consite/) software (Fig. 1B). Alignment of the region (−101 bp to +1 bp) between human, rhesus, and mouse showed that the binding site of SRY at –11 bp was more conserved than the site at −94bp. We hypothesized that these two sites might be SRY binding sites.

To isolate the 5′-flanking region of the WDR5 gene containing the two SRY binding sites, a 249-bp fragment of genomic DNA (−134 to +115 of the WDR5 gene) was amplified and subcloned into a pGL3-basic vector. In order to test whether SRY could function on this reporter construct, the reporter construct was cotransfected with either an SRY expression plasmid (pCXN-2-SRY-3HA) or an empty plasmid (pCXN-2) into human prostate adenocarcinoma LNCaP cells, which have very low expression levels of endogenous SRY, and relative luciferase activity was analyzed. Compared to empty vector, luciferase activity was increased 3.6-fold when cells were transfected with SRY expression plasmid (Fig. 1C). To verify that the SRY binding elements functioned for activation of human WDR5 expression, we constructed three reporter plasmids in which point-mutations were introduced into one or both SRY binding sites, and tested luciferase activity. Luciferase activity from mutants in which either SRY binding element was mutated was significantly reduced compare to the wild type. Moreover, luciferase activity from a mutant in which both SRY binding sites were mutated was further reduced (Fig. 1D). These data indicated that these two SRY binding sites contributed to the transcriptional activity of human WDR5.

### SRY binds to the promoter of WDR5

**Figure 2:**
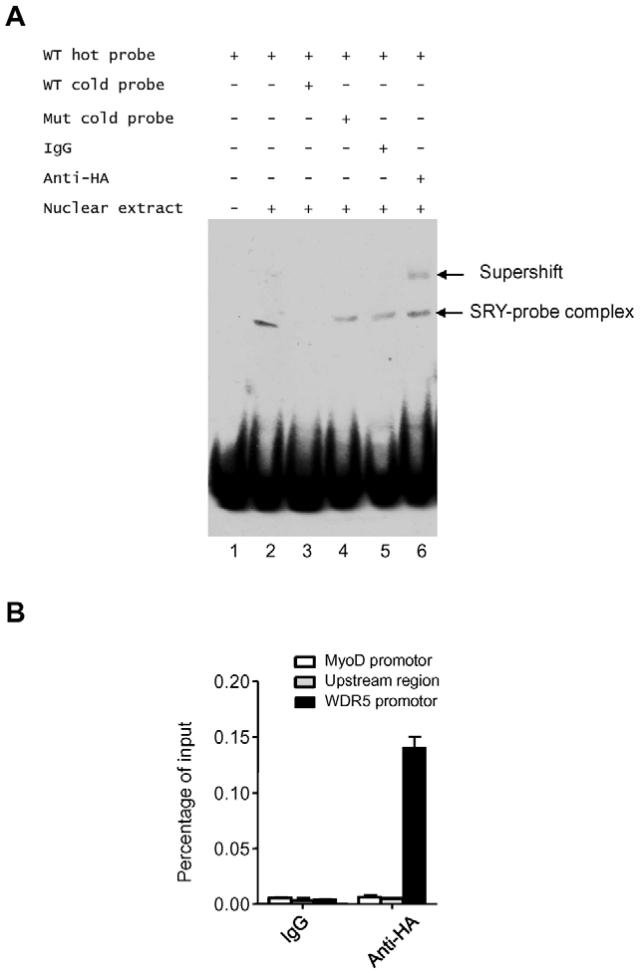
SRY binds to WDR5 promoter. (A) EMSA analysis of HA tagged SRY with wild type and mutant probes. Arrows indicate SRY-probe complex and supershift. (B) ChIP analysis of HA tagged SRY on WDR5 promoter,WDR5 promoter proximal upstream region, or MyoD promoter. Mouse IgG serves as a negative control. Graphs show mean ± SD, n = 3.

To determine if SRY bound the WDR5 promoter directly, electrophoretic mobility shift assays were performed. A wild type labeled probe bound the nuclear extract from transfected LNCaP cells overexpressing HA-tagged SRY (Fig. 2A, lane 2). This binding was ablated by competition with unlabeled wild type probe, but not by competing mutant probe (Fig. 2A, lanes 3, 4). A supershift band was observed when anti-HA antibody was added in nuclear extract incubated with wild type labeled probe, but not with control IgG (Fig. 2A, lanes 5, 6). This experiment indicated that SRY bound the WDR5 promoter in vitro.

Because of a lack of a high quality ChIP-grade antibody against human SRY, we performed ChIP assays on LNCaP cells overexpressing HA-tagged SRY, as above, to confirm that SRY associated with the endogenous WDR5 promoter. The results demonstrated that precipitation of HA-SRY brought down the WDR5 promoter, but did not bring down a control region upstream of the WDR5 promoter, or a negative control promoter, MyoD (Fig. 2B). Taken together, results of both EMSA and ChIP assays indicated that SRY binds WDR5 promoter.

### SRY activates WDR5 expression

**Figure 3:**
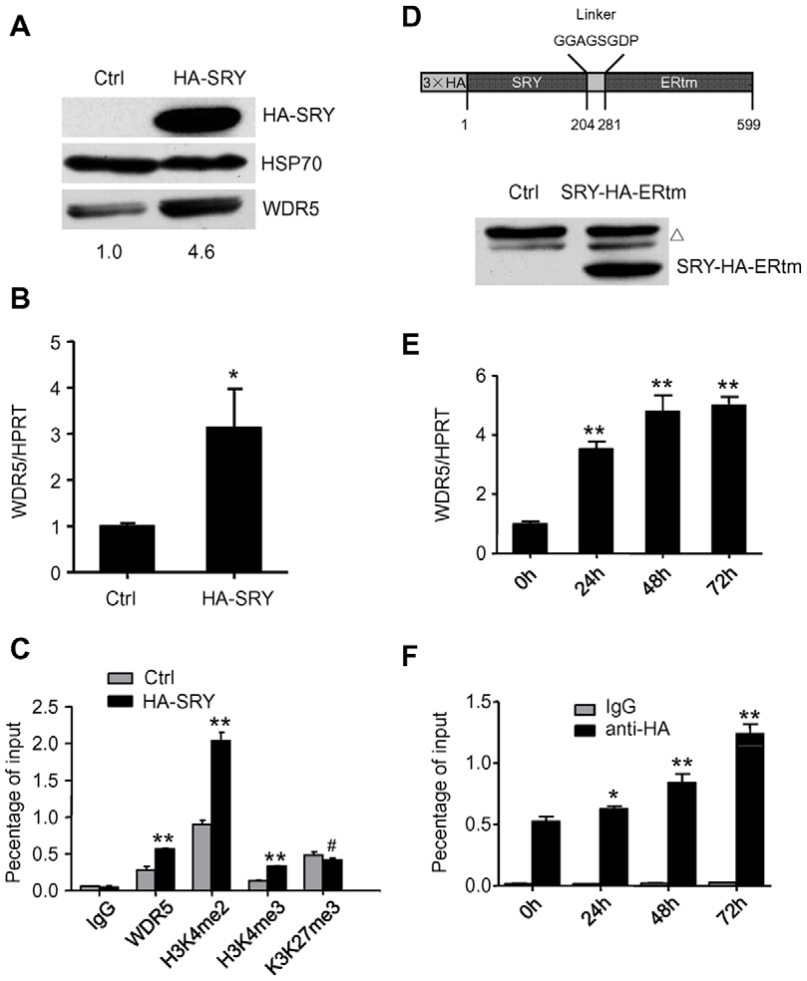
SRY activates WDR5 expression. (A) Western blot analysis of HA tagged SRY, Hsp70, and WDR5 from LNCaP cells containing either control vector (Ctrl) or HA-tagged SRY. Relative quantitation of WDR5 protein with ImageJ software (NIH, USA) is shown on the bottom. (B) Quantitative real-time analysis of WDR5 levels as in (A). Graphs show mean ± SD, n = 3. (C) ChIP analysis on WDR5 promoter with indicated antibodies as in (A). Graphs show mean ± SD, n = 3. (D) Schematic representation of HA-SRY-ERtm construct. Numbers show the respective amino acid positions of the individual constituents (upper panel). Western blot analysis of SRY-HA-ERtm expression with anti-HA antibody. A triangle indicates non-specific bands (lower panel). (E) Quantitative real time analysis of WDR5 levels from cells with SRY-3HA-ERtm after induction by 4-OHT at indicated time points. Graphs show mean ± SD, n = 3. (F) ChIP analysis on WDR5 promoter with either IgG or anti-HA antibody as in (E). Graphs show mean ± SD, n = 3. *P<0.05, **P<0.01, #P>0.05 compared to control; Student's t-test.

In order to assess the effect of SRY on endogenous protein, both WDR5 mRNA and protein expression levels were analyzed in LNCaP cells stably overexpressing HA tagged SRY. Consistent with reporter gene assays, overexpression of SRY induced endogenous WDR5 expression as shown in Western blot assay with anti-WDR5 antibodies (Fig. 3A). To confirm this result, we performed real-time PCR using specific primer for WDR5 cDNA. We found that the WDR5 mRNA level was three-fold higher in SRY overexpressing cells than control (Fig. 3B). In order to examine changes in epigenetic histone modification marks, we performed ChIP analyses of the WDR5 gene promoter with antibodies to H3K4me2, H3K4me3, and H3K27me3. Consistent with the expression data, histone H3K4me2 and H3K4me3 on the WDR5 promoter were enriched whereas H3K27me3 remained unchanged. We observed that WDR5 binding to its own promoter was significantly increased as well (Fig. 3C).

To further confirm that SRY activates WDR5 expression specifically, we established an inducible system using 4-OHT induction, and performed a time course assay. The ERtm domain (amino acids 281–599 of murine estrogen receptor with a Glycine to Arginine substitution at amino acid 525) was fused to the full length HA tagged SRY, separated by a glycine-rich linker (Fig. 3D). The modified ERtm domain is deficient in binding endogenous estrogen, but remains responsive to activation by the synthetic estrogen derivative 4-OHT or its precursor tamoxifen. Proteins fused to ERtm are retained outside the nucleus in a complex with heat shock proteins (Hsp), such as Hsp90. Upon binding to 4-OHT, the fusion protein is released and shuttled into the nucleus where it acts as a transcription factor [20]. The assembled cDNA was inserted into a murine stem cell virus (MSCV)-based retroviral vector MSCV-IRES-GFP, yielding MSCV-SRY-HA-ERtm-IRES-GFP. Using FACS sorting, we generated a stable LNCaP cell line overexpressing SRY-HA-ERtm. Expression of SRY-HA-ERtm was determined by immunoblotting with a monoclonal antibody against HA (Fig. 3D). LNCaP cells stably overexpressing SRY were harvested at different times after 4-OHT treatment. RNA from these cells was isolated and analyzed by quantitative real time PCR, which revealed that WDR5 mRNA levels were significantly increased after 24 hours induction (Fig. 3E). Consistent with this, anti-HA antibody ChIP experiments indicated that HA-tagged SRY was increasingly enriched on the WDR5 promoter following 4-OHT treatment (Fig. 3F). This result suggested that the accumulated SRY on the WDR5 promoter could be directly activating WDR5 expression.

### WDR5 regulates itself through a feedback loop

**Figure 4:**
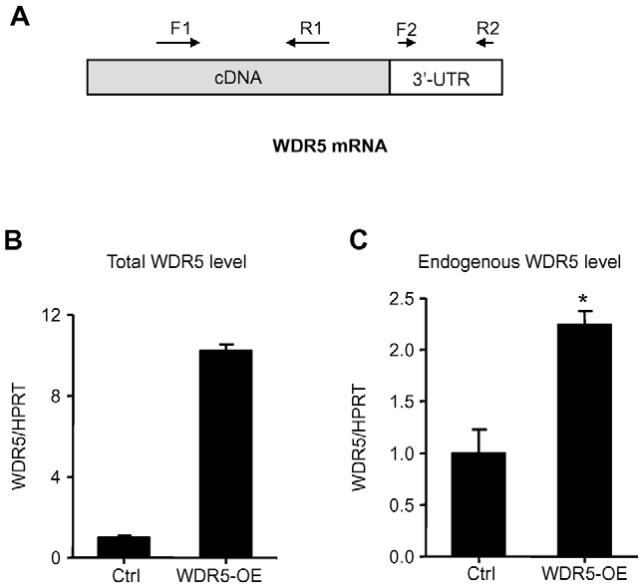
WDR5 regulates itself through positive feedback loop. (A) Schematic representation of primers to detect total (F1 and R1) and endogenous (F2 and R2) WDR5 mRNA. (B) Total WDR5 mRNA levels from cells containing either control vector (Ctrl) or overexpressing WDR5. Graphs show mean±SD, n = 3. (C) Endogenous WDR5 mRNA levels as in (B). Graphs show mean ± SD, n = 3. *P<0.05 compared to control; Student's t-test.

Since WDR5 binds its own promoter (Fig. 3C), we investigated whether WDR5 regulated its own expression. To test this, we generated a stable LNCaP cell line which overexpressed WDR5 protein exogenously. Endogenous mRNA can be distinguished from total mRNA by real time-RT-PCR using two different pairs of primers (Fig. 4A). Q-RT-PCR analysis revealed that the endogenous WDR5 mRNA level was increased 2-fold compare to the control, while the total WDR5 mRNA level was increased about 10-fold compare to the control (Fig. 4B, 4C). These results indicated that WDR5 could activate itself through positive feedback.

### SRY cooperates with WDR5 to induce Sox9 expression and repress β-catenin expression

**Figure 5:**
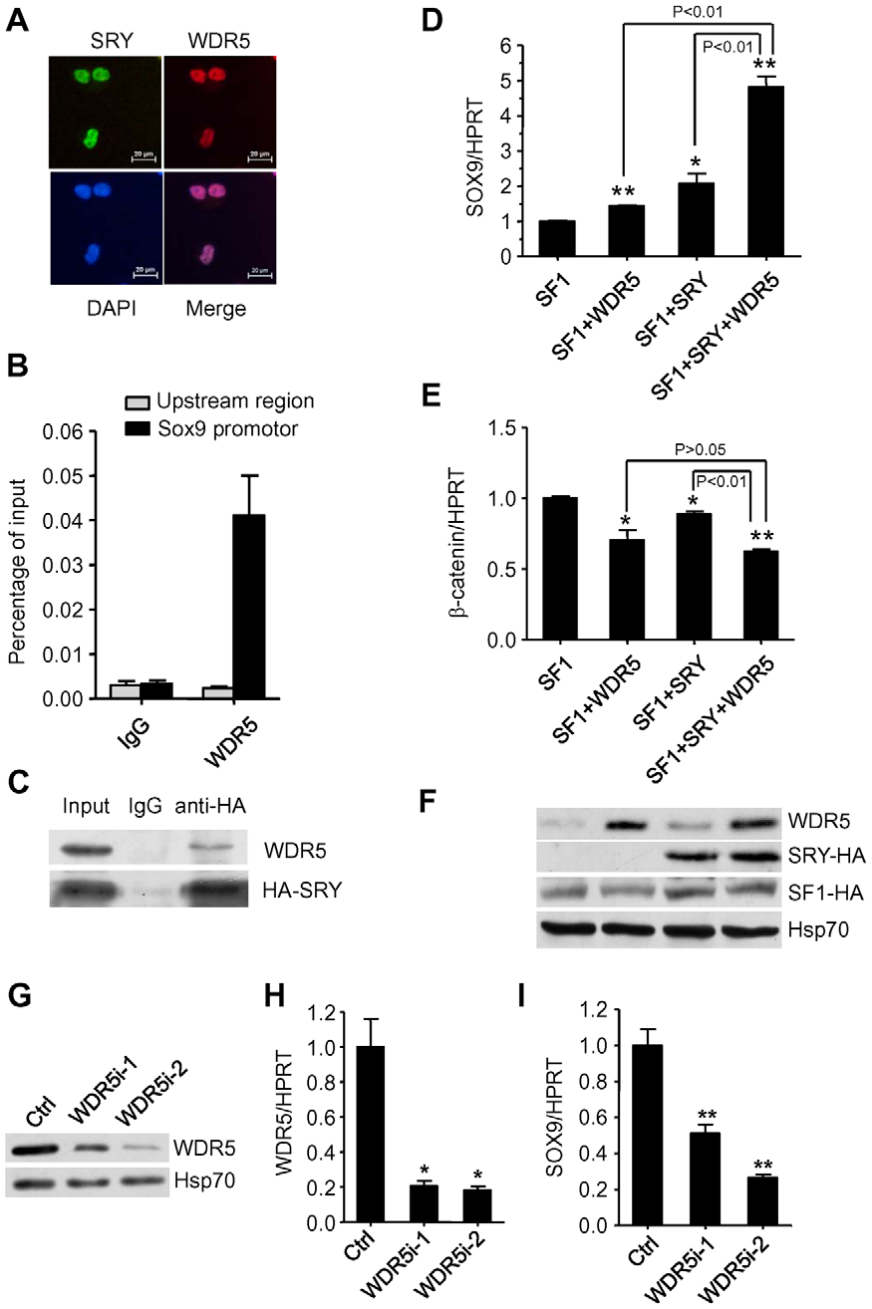
SRY cooperates with WDR5 to induce Sox9 expression. (A) Immunofluorescence analysis of HA-tagged SRY and WDR5 in LNCaP cells. (B) ChIP-reChIP (anti-HA antibody ChIP followed by anti-WDR5 antibody ChIP) analysis of HA-tagged SRY and WDR5 on Sox9 promoter, or Sox9 promoter proximal upstream region. Graphs show mean ± SD, n = 3. (C) Coimmunoprecipitation of WDR5 and HA-tagged SRY from LNCaP cells. (D) Quantitative real time PCR analysis of Sox9 levels from LNCaP cells containing SF1, SF1+WDR5, SF1+SRY, or SF1+SRY+WDR5. Graphs show mean ± SD, n = 3. *P<0.05, **P<0.01 compared to SF1 control; Student's t-test. (E) Quantitative real time PCR analysis of β-catenin levels as in (D). Graphs show mean ± SD, n = 3. *P<0.05, **P<0.01 compared to SF1 control; Student's t-test. (F) Western blot analysis of cellular lysate from cells as in (D). (G) Western blot analyses of cellular extracts from WDR5i-1 and WDR5i-2 or scrambled control (Ctrl) LNCaP cells with indicated antibodies. (H) WDR5 gene expression analysis by Q-RT-PCR of RNA from WDR5i-1, WDR5i-2 and scrambled control (Ctrl) LNCaP cells. Graphs show mean ± SD, n = 3. *P<0.05 compared to the scrambled control, Student's t-test. (I) Sox9 gene expression analysis by Q-RT-PCR of RNA from WDR5i-1, WDR5i-2 and scrambled control (Ctrl) LNCaP cells. Graphs show mean±SD, n = 3. **P<0.01 compared to the scrambled control, Student's t-test.

Next, we examined whether WDR5 could interact with SRY in vivo. To test this, we performed immunofluorescent staining experiments. The results showed that WDR5 co-localized with SRY in the nucleus of LNCaP cells (Fig. 5A). In addition, a ChIP-reChIP (anti-HA followed by anti-WDR5) experiment demonstrated that SRY and WDR5 interacted on the Sox9 promoter, but not a control region upstream of the promoter (Fig. 5B). The interaction between WDR5 and SRY was verified by co-immunoprecipitation experiments in which cellular extracts from LNCaP cells overexpressing SRY-3HA were co-immunoprecipitated with anti-HA antibody and blotted with anti-WDR5 antibody (Fig. 5C). SRY was expected to recruit more MLL complex components rather than to disrupt the complex (Fig. S2).

SRY is the critical gene that initiates male sex determination in most mammals. The best direct target for SRY is Sox9. In order to determine whether SRY or WDR5 can regulate Sox9 expression, we established LNCaP cell lines which expressed either SRY, WDR5, or both together with the nuclear orphan receptor, steroidogenic factor (SF1). Real time PCR results indicated that SRY and WDR5 together activated the expression of Sox9 significantly more than either alone (Fig. 5D). Consistent with their function in triggering male sex determination, SRY and WDR5 significantly reduced the expression levels of β-catenin, which is important for development of ovaries (Fig. 5E). Expression of WDR5, HA tagged SRY, and HA tagged SF1 was confirmed by Western blot (Fig. 5F). To further probe the direct role of WDR5 alone on Sox9 expression, we generated two WDR5 knockdown (WDR5i-1 and WDR5i-2) LNCaP cell lines using specific short hairpin RNAs, and a scrambled control line. In the knockdown lines, WDR5 levels were reduced to about 20% of the scrambled cells (Fig. 5G, 5H), and Q-RT-PCR demonstrated a 20-45% decrease in Sox9 expression in these cells compared to control cells (Fig. 5I). In aggregate, these results suggest that WDR5 together with SRY could play an important role in sex determination.

### Localization of the WDR5 protein and expression of the WDR5 gene in the murine embryonic testis

**Figure 6:**
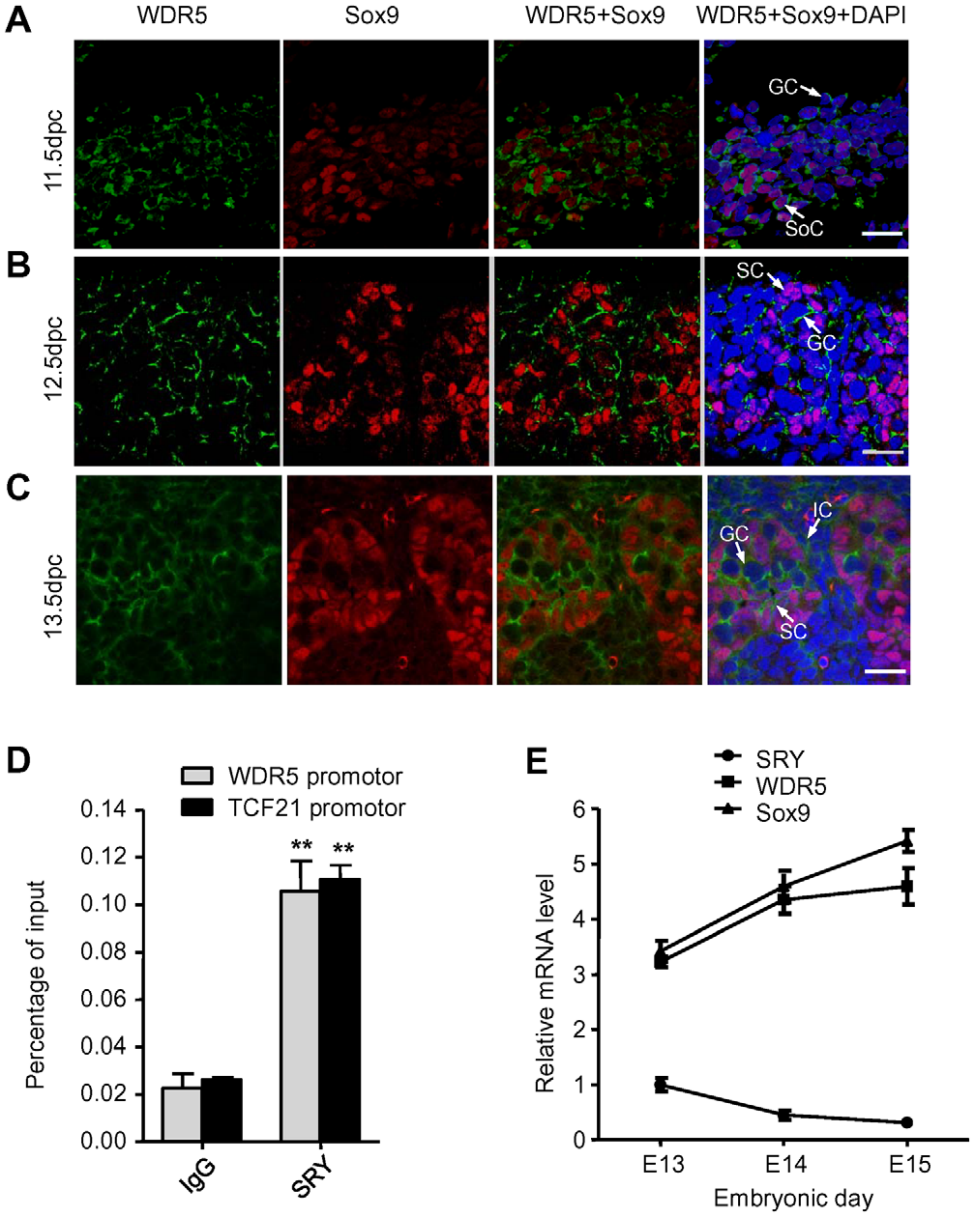
Immunolocalization of WDR5 protein and gene expression in embryonic testis. (A) Immunofluorescent staining of WDR5 (in green) and Sox9 (in red) protein in mouse E11.5 testis. Soc, Somatic cells; GC, Germ cells. Scale bar, 20 µM. (B) Immunofluorescent staining of WDR5 (in green) and Sox9 (in red) protein in mouse E12.5 testis. SC, Sertoli cells; GC, Germ cells. Scale bar, 20 µM. (C) Immunofluorescent staining of WDR5 (in green) and Sox9 (in red) protein in mouse E13.5 testis. IC, interstitial cells. Scale bar, 20 µM. Data shown are representative of three independent experiments. (D) ChIP analysis of SRY on rat WDR5 promoter and Tcf21 promoter. Graphs show mean ±SD, n = 3. **P<0.01 compared to control; Student's t-test. (E) Quantitative real time PCR analysis of WDR5, Sox9, and SRY levels relative to β-actin in male rat gonads at indicated embryonic stages.

To study the expression pattern of WDR5 in mouse embryonic testis, timed-pregnant mice were set up. Genital ridges from different days of these mice were collected and sectioned for immunofluorescent analysis with specific antibodies. At 11.5 dpc (days post-coitum), WDR5 was localized to germ cells and co-localized with Sox9 in somatic cells (Fig. 6A). At 12.5 dpc, WDR5 immunopositive cells were found predominantly in germ cells and occasionally in Sertoli cells (Fig. 6B). At 13.5 dpc, WDR5 was detected in germ cells, Sertoli cells, and in the interstitium (Fig. 6C). Of note, WDR5 was mostly localized in the cytoplasm rather than in the nucleus. It has previously been reported that WDR5 is more abundant in the cytoplasm than in the nucleus of human embryonic kidney 293 cells, and that it can be translocated from the nucleus to the cytoplasm during viral infection [21]. However, the movement of WDR5 between nucleus and cytoplasm in developing testes is currently unknown. The differential localization of WDR5 at different stages of development suggested different roles and functions for WDR5 in testis differentiation and sex determination. In order to confirm whether SRY binds the WDR5 promoter in vivo, ChIP analysis was performed from E13 rat gonads. The results demonstrated significant enrichment of SRY on the WDR5 promoter, similar to SRY binding on the Tcf21 promoter, a positive control (Fig. 6D). Moreover, Q-RT-PCR revealed that WDR5 and Sox9 displayed a similar expression profile in rat gonads from E13 to E15 (Fig. 6E). In aggregate, these results indicted that WDR5 and SRY are likely to have direct regulatory effects on Sox9 expression.

## Discussion

Although WDR5 is a core subunit of MLL/SET1 complexes, it also functions as a subunit of other complexes. WDR5 has been shown to be important in bone morphogenesis, vertebrate development, and embryonic stem cell renewal [4]–[6]. However, a function of WDR5 in sex determination has not been previously reported. In this study, we demonstrated that WDR5 is a direct target of SRY. In addition, WDR5 can regulate itself through a positive feedback loop. Furthermore, WDR5 can synergize with SRY to activate Sox9 while repressing the expression of β-catenin. All these results suggest that WDR5 may have an important role in sex determination.

Since the SRY gene was discovered in 1990, an answer to the question as to how SRY promotes testis differentiation has remained elusive [7], [22], [23]. Although some pathways regulating sexual differentiation have been elucidated, the details of SRY function were poorly understood. Currently, the dominant theory is that SRY plays a critical role in early gonad development in either direction (male or female) by pushing the balance to favor the male development pathway [18], [19]. In this process, Sox9, which is regulated by SRY, controls Sertoli cell formation and, consequently, testis differentiation. In our experiment, we showed that SRY can directly regulate WDR5 expression. The expressed WDR5 subsequently acts together with SRY to promote Sox9 expression.

The auto feedback loop involved in the regulation of WDR5 is interesting. During testis differentiation, many feedback loops have been observed. In mouse, SRY is expressed for only a short developmental period (dpc10.5–12.5) [24], [25], but Sox9 expression needs to be maintained for testis formation. Sox9 has been shown to contribute to its own expression [14]. In addition, a target gene of Sox9, Fgf9, also displays a positive feedback loop in which its expression helps to activate Sox9 [26]. In contrast to Fgf9, WDR5 seems to act upstream of Sox9. This may be a common scenario of how sex differentiation is achieved.

Most studies of mammalian sexual determination have been carried out using mouse models. In mice, at dpc10, genital ridges are formed without morphological differences between male and female [18]. At this time in male differentiation, SRY begins to be expressed and triggers expression of other genes involved in the differentiation of Sertoli cells. Female-specific gene expression leading to differentiation of granulose cells must be repressed [19]. β-catenin, a signature gene in ovary development, needs to be repressed [27]. This is consistent with our results that SRY and WDR5 together not only increase Sox9 expression, but also repress β-catenin expression.

The question arises, how can SRY and WDR5 play opposite roles on different genes? There is little doubt that different transcription factors or epigenetic modifiers and co-factors recruited by SRY and WDR5 must help to determine gene activity. In fact, histone H3K4 methylation, which is usually associated with WDR5, has been shown to be a dual factor. Although H3K4 methylation is largely associated with transcription initiation and elongation [2], [28], some evidence indicated that this mark could also be involved in gene repression. In yeast, H3K4me2/3 induced by Set1 can directly contribute to repressive machinery on PHO5 and PHO84 genes [29], [30]. H3K4me3 can also be recognized by ING2, a component of the Sin3-HDAC complex, to repress the Cyclin D gene in mammalian cells [31]. However, we could not exclude the possibilities that WDR5 and SRY recruit different co-factors to act on different genes.

In summery, we have identified WDR5 as a novel direct target of SRY. More interestingly, WDR5 can further cooperate with SRY to regulate Sox9 and β-catenin expression. It would be interesting to determine the molecular mechanism by which SRY plays a dual role at the early stage of mammalian sex determination. The finding that WDR5 cooperates with SRY will help probe their roles in testis differentiation.

## Materials and Methods

### Antibodies and reagents

Anti-HA (12CA5) antibody was purchased from Roche. Anti-WDR5, anti-H3K4me2, and anti-H3K27me3 antibodies were purchased from Abcam. Anti-H3K4me3 antibody was purchased from Millipore. Anti-Hsp70 and anti-SRY (mouse) antibodies were purchased from Santa Cruz. The pGL3-Basic luciferase vector was purchased from Promega. DMEM, RPMI 1640, and fetal bovine serum were obtained from Life Technologies.

### Cell lines

LNCaP cells (gifted by Dr. Jiemin Wong, East China Normal University, China) were seeded in a 100-mm dish and transfected with 10 μg pCXN-2-SRY-3HA plasmid when the confluency reached 80%; 36 hours after transfection, cells were supplied with fresh media containing 600 µg/ml G418. Resistant clones were selected within 15–20 days and expanded. The G418 concentration was maintained at 400 µg/ml in the cell culture medium.

The WDR5 coding regions and SRY-3HA-ERtm sequence were cloned into the retroviral vector plasmid 3HA-MSCV-IRES-GFP at unique XhoI or EcoRI sites and amphotropic viral supernatant was obtained as described previously [32].

The siRNA target sequences for WDR5 were inserted into the *Xho*I/*Hpa*I sites in the pLL3.7 lentiviral vector according to the manufacturer's recommendations (American Type Culture Collection, USA). The oligonucleotides are:

WDR5i-1 sequences: GTGGAAGAGTGACTGCTAA;

WDR5i-2 sequences: GAATGAGAAATACTGCATA

Retroviral supernatant was filtered and added to LNCaP cells every 24 hours for 3 days. Lentivirus production in 293T cells and infection of LNCaP cells were performed as described previously [33]. Cells expressing GFP were sorted by sterile flow cytometry and expanded.

### Site-directed mutagenesis and luciferase reporter assay

Genomic DNA was extracted from 293T cells as described previously [34]. A fragment (−134 to +115) containing the proximal promoter of WDR5 was amplified by PCR using the following primers: sense, 5′-GCGGTACCAGGACTTAGGGGAATTAATAG -3′, which contained a KpnI restriction site and antisense, 5′-CGCAGATCTGTCTCGGGCTTCTTCTC-3′, which contained a BglII restriction site. The PCR product was cloned into the pGL-3 vector. Mutations were obtained using a site-directed mutagenesis kit (SBS technologies, Shanghai). All mutated insert fragments were confirmed by sequencing.

For the luciferase reporter assay, 3×10^4^ LNCaP cells were plated in 12 well plates 24 hours prior to transfection. Triplicate wells were transiently transfected with the indicated plasmids using lipofectamine 2000 (Life technologies), and 36 hours after transfection, relative luciferase activity was measured using the Luciferase Reporter Assay System (Promega). Beta-galactosidase assays were performed as normalization controls according to the Cold Spring Harbor Protocol [35]. β-galactosidase enzyme activity was measured using a Universal Microplate Spectrophotometer.

### Co-immunoprecipitation, immunofluorescence, and histology

For co-immunoprecipitation studies, nuclear extracts were extracted from LNCaP cells and incubated with HA or WDR5 antibodies for 2 hours at 4°C. A 50% slurry of protein G Sepharose was added and incubated overnight at 4°C. The mixture was then centrifuged and the pellet was washed 4 times in 50 mM Tris-HCl, pH 7.9, containing 150 mM NaCl prior to being resuspended in SDS loading buffer. Samples were separated by SDS-PAGE, immunoblotted, and probed with the relevant antibodies. Western blots were detected by using an ECL kit according to the instructions of the manufacturer (Thermo Scientific).

For immunofluorescence, LNCaP cells overexpressing SRY-3HA were mounted on polylysine slides and fixed in 4% paraformaldehyde for 30 min. After being permeabilized with 0.1% (v/v) Triton X-100, cells were blocked in PBS containing 10% goat serum for 30 min at room temperature. The cells were then incubated with the polyclonal anti-rabbit WDR5 and anti-mouse HA primary antibodies at 4°C overnight. After washing cells four times with PBS, secondary antibodies, goat TexasRed anti-rabbit IgG and FITC anti-mouse IgG (Vector Laboratories) were applied in PBS for 1 h at room temperature. The slides were washed and counterstained with 4,6-diamidino-2-phenylindole (DAPI) for 3 min before imaging with a Nikon Eclipse 80i microscope (Nikon).

For mouse histology and immunofluorescence analysis, genital ridges from timed-pregnant mice were collected at E11.5, E12.5, and E13.5. Sex was determined for Sry on genomic DNA from embryo tails by standard PCR. Animal studies were approved by the Animal Care and Use Committee of the Model Animal Research Center, the host for the National Resource Center for Mutant Mice in China, Nanjing University. Genital ridges were fixed in 4% paraformaldehyde and dehydrated through 30% sucrose solution two hours later. Fixed tissues were embedded in Jung Tissue Freezing Medium, and serially sectioned. Sections were washed three times in PBS,transferred to blocking solution containing 5% donkey serum in 0.1% Tween/PBS for 1 hour, and incubated with primary antibody at 4°C overnight. After washing with PBS, the secondary antibody was added and sections were incubated for 2 hours at room temperature. Slides were mounted and analyzed with a BX51 Olympus fluorescence microscope connected to a DP 20 digital camera (Olympus Corporation, Japan). Combinations of the first and secondary antibody were as follows: rabbit anti-Sox9 (1∶50; Santa Cruz, sc-20095) and goat anti-WDR5 (1∶50; R&D, AF5810); FITC donkey anti-goat IgG (1∶200; Abcam, ab6881) and Alexa Fluor 594 donkey anti-rabbit IgG (1∶200; Invitrogen, A21207).

### RNA isolation and Real time-PCR

RNA was isolated from cells with Trizol reagent (Life Technologies) according to the manufacturer's protocol. cDNA was synthesized with the SuperScript first-strand synthesis system (Life Technologies). Q-RT-PCR primers are provided as following. Real-time quantitative RT-PCR was performed using the FastStart Universal SYBR Green Master (Roche) in a Rotorgene 6000 (Corbett Research) in a final volume of 20 µl. Cycling conditions were 94°C for 15 s, 60°C for 30 s and 72°C for 30 s. Each reaction was done in triplicate.

The primers for human hypoxanthine guanine phosphoribosyltransferase (HPRT) were:

forward 5′-ATGGACAGGACTGAACGTCT,

reverse 5′-CTTGCGACCTTGACCATCTT.

The primers for human WDR5 were:

forward 5′-CACAAGCTGGGAATATCCGATG,

reverse 5′-GGGGATTGAAGTTGCAGCAAAA.

The primers for human WDR5 (UTR) were:

forward 5′-CGAGAGACTGTCGGGAAGTTG,

reverse 5′- TCCCTAGACAGTGTTAGAAT.

The primers for human Sox9 were:

forward 5′-TACGACTGGACGCTGGTG,

reverse 5′-TCTCCAGAGCTTGCCCAGCGT.

The primers for human β-catenin were:

forward 5′-GAAACGGCTTTCAGTTGAGC,

reverse 5′-CTGGCCATATCCACCAGAGT

The primers for rat WDR5 were:

forward 5′-CGTGAGTTCCGGAAAGTGTCTGAAG,

reverse 5′-GAAATGAACGGCTGAGACTGGAT

The primers for rat Sox9 were:

forward 5′-TGAAGATGACCGACGAGCAGGAGAAG,

reverse 5′-CTTCCTCGCTCTCCTTCTTCAG

The primers for rat SRY were:

forward 5′-CATCGAAGGGTTAAAGTGCCA,

reverse 5′-ATAGTGTGTAGGTTGTTGTCC

The primers for rat β-Actin were:

forward 5′-GTCGACAACGGCTCCGGCA,

reverse 5′-AGGTCTCAAACATGATCTGGGT

### Electrophoretic mobility shift assays (EMSA)

To assess the DNA binding activity of SRY in vitro, EMSA was performed. Nuclear extracts were prepared from LNCaP cells overexpressing SRY-3HA as described previously [36]. EMSA was performed by using a LightShift EMSA optimization and control kit (Pierce, Rockford, USA). The double-stranded oligonucleotides correspond to the sequence −15 to +9 (5′-biotin-ATAGTTTGTTTCTTGGCTCCCTGT-3′) of the WDR5 promoter region. For the binding reaction, 20 fmol biotin-labeled, double-stranded oligonucleotides were incubated with nuclear extract (2–5 μg) in 1× binding buffer, 1 μg poly-dI:dC, 20 min at room temperature. For competition studies, unlabeled wild-type or mutant double-stranded oligonucleotides (50-fold molar excess) were pre-incubated with nuclear extract before addition of labeled oligonucleotides. For supershift assays, extracts were preincubated with 2 µg mouse IgG or anti-HA antibody for 15 min at room temperature before addition of the probe. Reaction products were separated in 6.5% native polyacrylamide gels in 0.5× TBE buffer and visualized using the LightShift EMSA kit (Pierce).

### Chromatin Immunoprecipitation (ChIP) Assays

ChIP assays were performed as described previously [33], [37]. For re-ChIP experiments, immunoprecipitates from the single ChIP were eluted by incubation for 30 min, 37°C in 25 μl 10 mM dithiothreitol. The supernatant was removed, diluted at least 70 times using ChIP dilution buffer (1% Triton X-100, 2 mM EDTA, 150 mM NaCl, 20 mM Tris-HCl [pH 8.1]) and then subjected to another round of immunoprecipitation. PCR amplification or realtime PCR was performed using the purified DNA from either the single ChIP or the re-ChIP. For the in vivo ChIP, carrier ChIP (cChIP) analysis was adopted form Bhandari et al. [16]. Ten 13dpc rat gonads were used for the assay.

The ChIP primers for human WDR5 promoter were:

forward 5′-CTGCTGCATTCTTACAGACTTCTGG,

reverse 5′-TGACTACCATATTGAGCCCTGTAGC.

The ChIP primers for human WDR5 promoter proximal region were:

forward 5′-CCAGACCCACCAAGCCACTCAGT,

reverse 5′-GGAACGTAACCGCTCAAAATGGCT

The ChIP primers for human Sox9 promoter were:

forward 5′-ACCCTACCGTCCGCCCTTTG,

reverse 5′-CCGCCTCACCTTAGAGCCAC.

The ChIP primers for human Sox9 promoter proximal region were:

forward 5′-CATCTATTCGATCAGTCAACAG,

reverse 5′-CGCTGGGCTTGGAGAGTGTTTAT.

The ChIP primers for rat WDR5 promoter were:

forward 5′-GTCAGCCAGGCAGTTGAGAGTAC,

reverse 5′-AGCAGCCATCAGTCTCCCTCCAAT

The ChIP primers for rat Tcf21 promoter were:

forward 5′-TCTCCACACTGGTGATTAACAAA,

reverse 5′-TAATCCAGGCTCAGCTGAGA

## Supporting Information

Figure S1 **Diagram and alignment of WDR5 genes.** (A) Schematic representation of human WDR5 variants. Filled rectangles indicate exons, empty rectangles indicate UTR. (B) 2000 bp upstream of the two WDR5 variants from different vertebrates were aligned using the UCSC Genome Browser.
(TIF)

Figure S2 **Co-immunoprecipitation and Western blot analysis of MLL complex and SRY.** WDR5 antibody immunoprecipitates from vector-containing control (Ctrl) and SRY-3HA-overexpressing (SRY-OE) cells were blotted with indicated antibodies on the right.
(TIF)
