# Supplementary figures and images for: Synergistic Effect of SRY and Its Direct Target, WDR5, on Sox9 Expression
Source: PLoS One. 2012 Apr 16;7(4):e34327. doi: 10.1371/journal.pone.0034327 (PMC3327683; doi:10.1371/journal.pone.0034327)

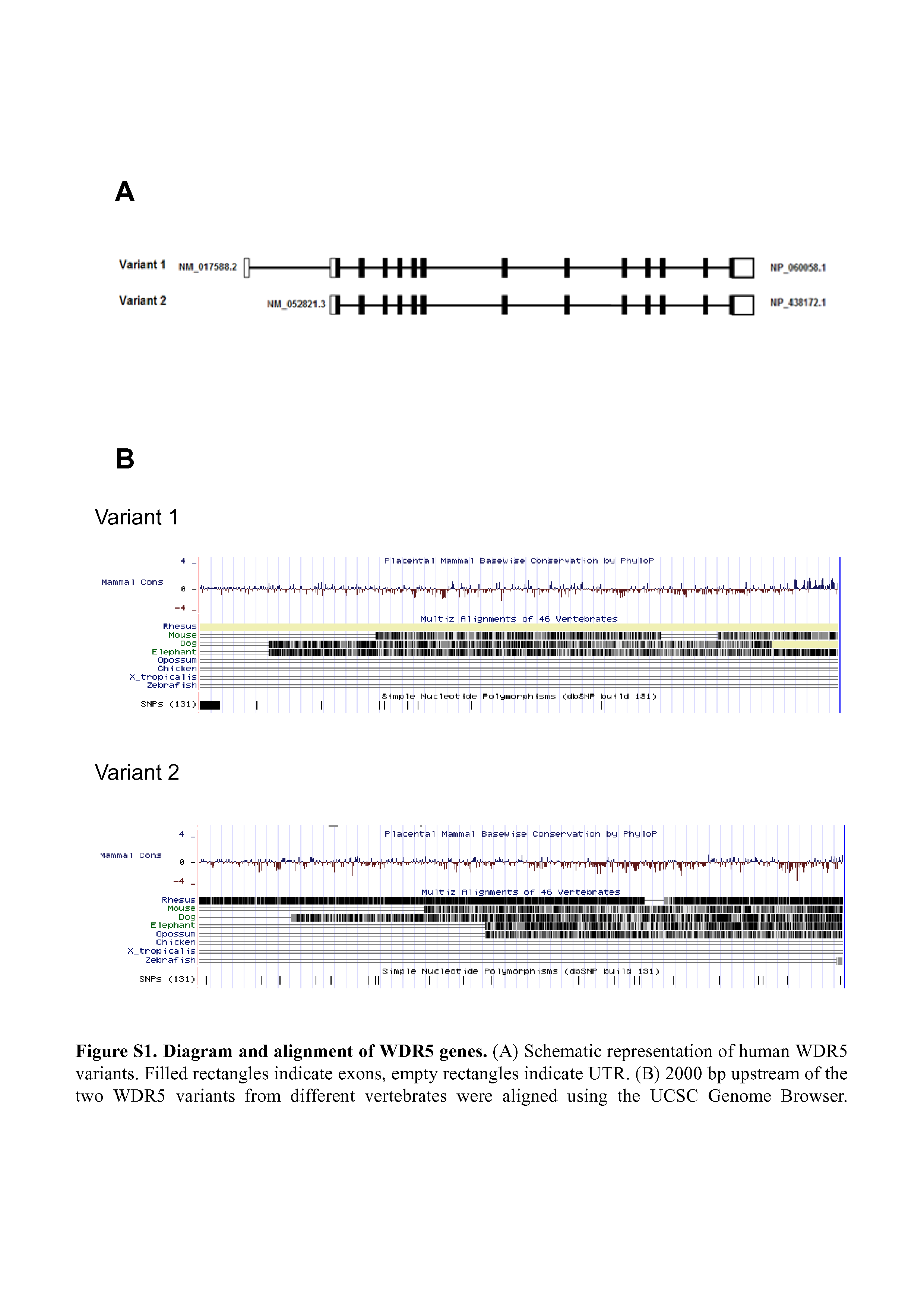

Supplement: Figure S1 — Diagram and alignment of WDR5 genes. (A) Schematic representation of human WDR5 variants. Filled rectangles indicate exons, empty rectangles indicate UTR. (B) 2000 bp upstream of the two WDR5 variants from different vertebrates were aligned using the UCSC Genome Browser. (TIF) [file pone.0034327.s001.tif]

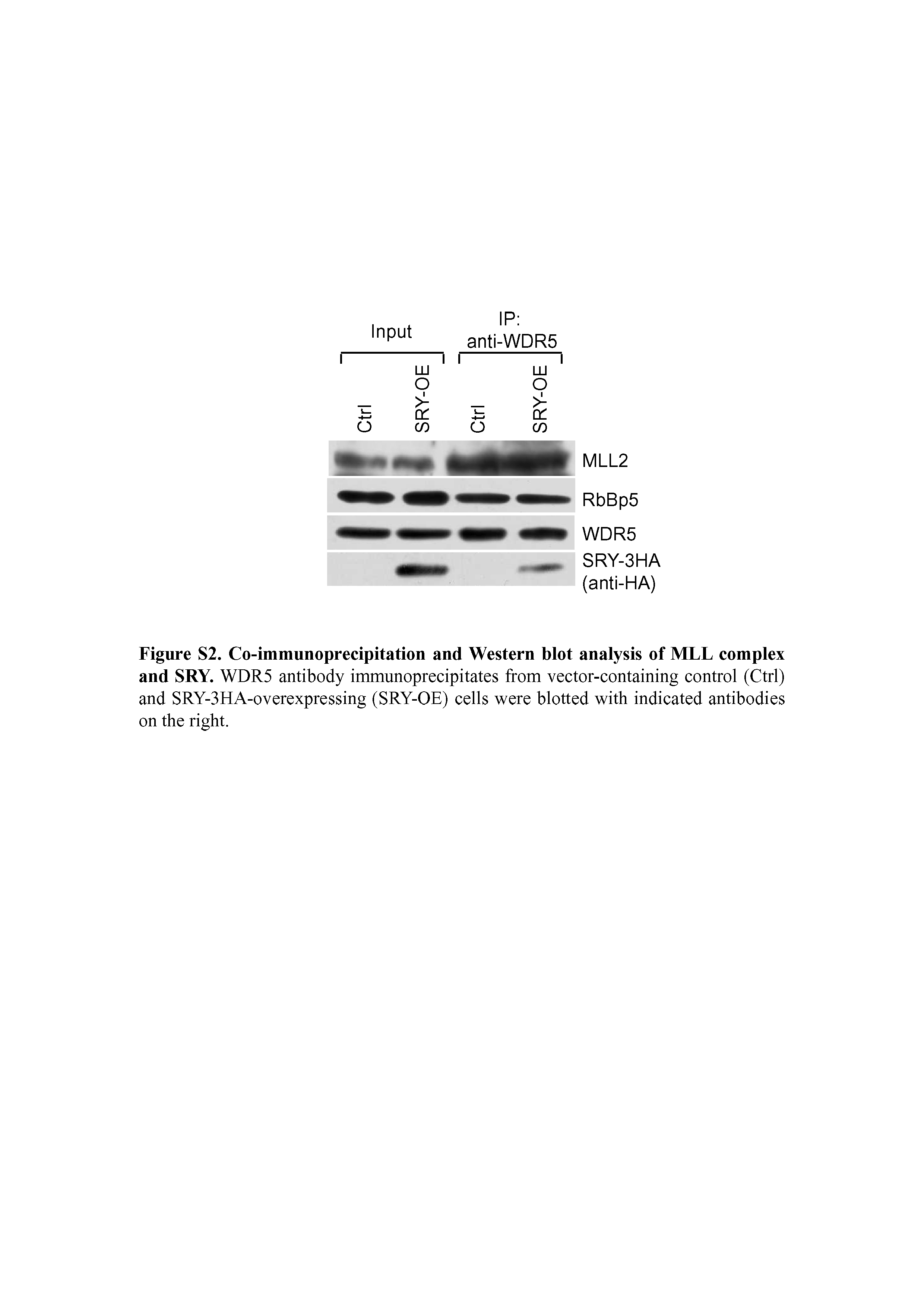

Supplement: Figure S2 — Co-immunoprecipitation and Western blot analysis of MLL complex and SRY. WDR5 antibody immunoprecipitates from vector-containing control (Ctrl) and SRY-3HA-overexpressing (SRY-OE) cells were blotted with indicated antibodies on the right. (TIF) [file pone.0034327.s002.tif]
